# *In Vitro* Antibacterial, Antioxidant, and Cytotoxic Activities of *Parthenium hysterophorus* and Characterization of Extracts by LC-MS Analysis

**DOI:** 10.1155/2014/495154

**Published:** 2014-05-07

**Authors:** Shashank Kumar, Sanjay Pandey, Abhay K. Pandey

**Affiliations:** ^1^Department of Biochemistry, University of Allahabad, Allahabad 211002, India; ^2^Instrumentation Division, Indian Institute of Integrative Medicine, Jammu 180001, India

## Abstract

Present work reports the biological activities of *P. hysterophorus* leaf, stem, flower, and root. Dried samples were sequentially extracted with many solvents. Hexane (HX), benzene (BZ), and chloroform (CH) extracts of leaf showed considerable antibacterial activity against *Streptococcus mutans* (MTCC 497), *Proteus vulgaris* (MTCC 7299), and *Salmonella typhi* (MTCC 3917). Flower extracts exhibited presence of higher amount of flavonoids (13.9–59.6 **μ**gQE/mg) followed by leaf, stem, and root. Stem (HX, BZ, and CH), leaf ethanol (ET), and root (HX, BZ, and CH) fractions showed noticeable antioxidant capacity in phosphomolybdate assay. Most of the extracts demonstrated beta carotene bleaching inhibition capability. BZ, ethyl acetate (EA), and ET fractions of leaves, stem aqueous (AQ), and flower EA extracts showed membrane protective activities (40–55%). Middle fractions of the plant parts displayed moderate antihemolytic potential. Most of the flower extracts exhibited cytotoxic activity (80–100%) against lung and colon cancer cell lines. Root (HX and ET) and leaf ET extracts showed considerable inhibition (90–99%) of colon and ovary cancer cell lines. The LC-MS scan demonstrated presence of different compounds showing 3–20 min retention time. The study revealed considerable antibacterial, antioxidant, lipo-protective, antihemolytic, and anticancer potential in all parts of *P. hysterophorus*.

## 1. Introduction

The imbalance in redox couples during metabolism such as those reduced to oxidized glutathione (GSH/GSSG) or NADPH/NADP^+^ ratios involve the overproduction of reactive free radicals which lead to a pathophysiological condition known as oxidative stress. The products of this imbalance are molecules that are enriched in one or more oxygen atoms (reactive oxygen species or ROS) that are generally considered to be markers of oxidative stress [1–3]. ROS are thought to be the major factors responsible for the alteration of cellular macromolecules, that is, proteins, DNA, and lipids. The reactive intermediates, produced by oxidative stress, can cause the peroxidation of polyunsaturated fatty acids. This in turn causes changes in the permeability and fluidity of the membrane lipid bilayer which can dramatically alter cell integrity. To combat ROS induced damage, cells possess several antioxidant enzymes and molecules [4]. It is well known that due to oxidative stress cancer initiation may take place and thus potent antioxidants show potential to combat progression of carcinogenesis. Potential of antioxidant as an anticancer agent depends on its competence as an oxygen radical inactivator and inhibitor. In UK around 159,000 people (252 deaths for every 100,000 people) died from cancer in the year 2011. The percentage of deaths from cancer is slightly higher in males (31%) than in females (27%), reflecting higher overall mortality rates in men [5]. There are reports that many types of cancer cells have increased levels of ROS which includes highly altered levels of antioxidant enzymes such as superoxide dismutase, glutathione peroxidase, and peroxiredoxin. Studies have also revealed increased levels of oxidized DNA base (8OHdG), the oxidative damage products in solid tumors [1, 2].

Phytoconstituents have been a source of medicine since time immemorial. Active phytochemicals can be derived from any part of plant like bark, leaves, flowers, roots, fruits, seeds, and so forth. Many effective anticancer and antioxidant agents in current use are derived from plants. Currently, over 50% of drugs used in clinical trials for anticancer activity were isolated from natural sources or are related to them [6, 7]. Flavonoids are a large group of polyphenolic compounds that have been shown to have antioxidative, hepatoprotective, cardioprotective, anti-inflammatory, antiviral, and anticancer activities. They are produced by plants as well as by genetically modified microbes [8].

Infectious diseases are responsible for large scale morbidity and mortality worldwide. For example* Salmonella typhi* causes typhoid fever and is exclusively adapted to infection of the human host. About 20 million cases of typhoid fever occur annually, resulting in approximately 600,000 deaths worldwide [9].* Streptococcus mutans* is associated with pyogenic and other infections in various sites including mouth, heart, and central nervous system [10]. The genus* Proteus* spp. is known to cause urinary tract infections. These are difficult to treat and usually associated with bladder and kidney stone formation that can lead to the obstruction of the urinary tract and catheters.* Proteus vulgaris* is one of the three species associated with urinary tract infections; others include* P. mirabilis* and* P. penneri* [11]. Treatment of infectious diseases with currently available drugs is associated with various side effects in addition to emergence of drug resistant bacterial strains [12]. Hence, it is imperative to discover novel antibacterial agents from natural sources that may have lesser side effects. Various studies have shown antimicrobial activities of the extracts prepared from variety of plants [13–15].

*Parthenium hysterophorus* L. (Asteraceae) is an invasive weed throughout the world commonly known as altamisa, carrot grass, bitter weed, star weed, white top, wild feverfew, and the congress grass. Plant has been used as folk remedy for the treatment of infectious and degenerative diseases [2, 4]. All parts of the plant are reported to be used as bitter tonic, febrifuge, emmenagogue, antidysenteric, and so forth. Some researchers have reported its use in traditional medicine for treatment of wounds, ulcerated sores, anemia, fever, and heart troubles [4]. In India and many other countries extracts of* P. hysterophorus* are used as ethno medicine against inflammatory, skin, neural, and female reproductive problems. In Maharashtra and Gujarat (India) the plant is used in the treatment of diabetes mellitus.* P. hysterophorus* has been found to be pharmacologically active as analgesic in muscular rheumatism and as vermifuge and therapeutic for neuralgia [16]. Present study reports the antibacterial, antioxidant, and cytotoxic activities of* P. hysterophorus* plant parts in addition to characterization of potent extracts by LC MS analysis.

## 2. Materials and Methods

### 2.1. Plant Material and Preparation of Extracts

The* P. hysterophorus* leaf, stem, flower, and root were shade-dried, crushed, and ground into fine powder with mortar and pestle. Powdered material was sequentially extracted with hexane (HX), benzene (BZ), chloroform (CH), ethyl acetate (EA), acetone (AC), ethyl alcohol (ET), and water (AQ) in Soxhlet apparatus as described earlier [17, 18]. The respective extract fractions were centrifuged, filtered, and lyophilized. The dried residues were dissolved in DMSO for determination of antibacterial, antioxidant, and anticancer activities.

### 2.2. Microorganisms and Growth Conditions

*Streptococcus mutans* (MTCC 497),* Proteus vulgaris* (MTCC 7299), and* Salmonella typhi* (MTCC 3917) were procured from the Institute of Microbial Technology, Chandigarh, India. The bacterial culture was maintained at 4°C on nutrient agar slants.

### 2.3. Evaluation of Antimicrobial Activity

Antimicrobial activity of plant extracts was determined using Kirby-Bauer disc diffusion method [19]. The inoculum suspension of bacterial strains was swabbed on the entire surface of Mueller-Hinton agar (MHA). Sterile 6 mm diameter paper discs (Himedia) saturated with 20 *μ*L of extracts prepared in DMSO (2 mg extract/disc) were aseptically placed on the upper layer of the inoculated MHA surfaces and plates were incubated at 37°C for 18 hours. Antibacterial activity was determined by measuring diameter of the zone of inhibition (ZOI) surrounding discs. Standard antibiotic discs meropenem (10 *μ*g/disc) and piperacillin tazobactam (100/10 *μ*g/disc) were used as positive controls. Discs containing 20 *μ*L DMSO were used as a negative control. Antimicrobial assay was performed in triplicates and results are reported as average of three replicates.

### 2.4. Quantitative Determination of Total Flavonoid Content

Aluminum chloride colorimetric method [20] as modified by us [21] was used for determination of flavonoids in various extract fractions of* P. hysterophorus* leaf, stem, flower, and root. Small amount (0.2 mL) of extract (2 mg/mL) in pure DMSO was separately mixed with 1.8 mL of methanol, 0.1 mL of 10% aluminum chloride, 0.1 mL of 1 M potassium acetate, and 2.8 mL of distilled water. Tubes were incubated at room temperature for 30 min and then absorbance of the reaction mixture was measured at 415 nm. The calibration curve was prepared with quercetin solution (1 mg/mL in methanol). Different volumes containing 20–200 *μ*g quercetin were taken in different tubes and volume was raised to 1.8 mL with methanol followed by addition of 0.2 mL DMSO. Rest of the procedure was same as described above. The amount of flavonoids in the test samples was expressed as *μ*g quercetin equivalent/mg sample (*μ*g QE/mg). Experiments were performed in triplicate and the results were expressed as mean ± SEM.

### 2.5. Phosphomolybdate Assay

The total antioxidant capacity of the extract fractions was determined by phosphomolybdate method using propyl gallate as standard [22] with some modification [21]. To 0.05 mL (100 *μ*g) of the extract solution prepared in DMSO, 0.25 mL methanol was added followed by the addition of 3 mL of reagent (0.6 M sulfuric acid, 28 mM sodium phosphate, and 4 mM ammonium molybdate). The tubes were capped and incubated in a water bath at 95°C for 90 min leading to development of green colour. After the samples had cooled to room temperature, the absorbance was measured at 695 nm. The total antioxidant capacity was expressed as *μ*g propyl gallate equivalents per gram of sample (*μ*g PGE/mg of sample) by using the standard graph. The results were expressed as mean ± SD (*n* = 3).

### 2.6. Beta-Carotene Bleaching Assay

The antioxidant activities of* P. hysterophorus* extracts were investigated according to the method described elsewhere [23]. Two gram of agar was completely dissolved in 100 mL hot water and the solution was allowed to cool to 50°C followed by addition of 4 mL linoleic acid (5 mg/mL in ethanol) and 20 mL of *β*-carotene (1 mg/mL in acetone). The agar was poured into Petri dishes and allowed to set for 30 min. Wells (4 mm diameter) were punched into the agar of each Petri dish using a sterile cork borer. Plant extracts (30 *μ*L) prepared in DMSO (2 mg/mL) was added to each well. BHT was used as standard. Plates were incubated overnight at 45°C until the background colour had bleached. The experiment was performed in triplicate and results are shown as mean ± SD.

### 2.7. *In Vitro* Lipid Peroxidation Inhibition Assay

Brain was isolated from normal albino Wistar rats and 10% (w/v) homogenate was prepared in phosphate buffer (0.1 M, pH 7.4 having 0.15 M KCl) using homogenizer (REMI motors Ltd., India) at 4°C. The homogenate was centrifuged at 800 g for 15 min and clear cell-free supernatant was used for the study. The lipo-protective efficacy of extracts in rat brain homogenate was estimated by the method of Halliwell and Gutteridge [24] with some modification [13]. 100 *μ*L extract solution containing 0.2 mg extract dissolved in respective solvents was taken in test tubes and evaporated to dryness followed by addition of 1 mL KCl (0.15 M) and 0.5 mL of brain homogenate. Peroxidation was initiated by adding 100 *μ*L FeCl_3_ (0.2 mM). After incubation at 37°C for 30 min, lipid peroxidation was monitored by the formation of thiobarbituric acid reactive substances (TBARS). TBARS were estimated by adding 2 mL of ice-cold hydrochloric acid (0.25 N) containing 15% TCA, 0.38% TBA, and 0.5% BHT. The reaction mixture was incubated at 80°C for 1 h and then cooled and centrifuged. The absorbance of the pink supernatant (malondialdehyde formed by the lipid peroxidation product and TBA complex) was measured at 532 nm. BHA was used as standard for comparison. All analyses were carried out in triplicate and results were expressed as mean ± SD. The protective effect of different extracts against lipid peroxidation (% LPOI) was calculated by using the following formula:

(1) $$\begin{array}{c} \text{LPOI}\left(\%\right)=\left[1-\frac{A_{S}}{A_{C}}\right]\times 100, \end{array}$$

where *A*_*C*_ is the absorbance of control and *A*_*S*_ is the absorbance of the standards or samples.

### 2.8. Antihemolytic Activity

Antihemolytic activity of the extracts was assessed by the method of Naim et al. [25]. The erythrocytes from rat blood were separated by centrifugation and washed with phosphate buffer (0.2 M, pH 7.4). The erythrocytes suspension (4%) was prepared in phosphate buffered saline. 50 *μ*L extract solution in DMSO (containing 100 *μ*g extract) was taken and volume was raised to 1.5 mL with saline buffer. It was added to 2 mL of the erythrocyte suspension. The mixture was incubated for 5 min at room temperature and then 0.5 mL of H_2_O_2_ solution in saline buffer was added to induce the oxidative degradation of the membrane lipids. The concentration of H_2_O_2_ in the reaction mixture was adjusted to produce about 90% hemolysis of blood cells after 225 min. After incubation the reaction mixture was centrifuged at 1500 rpm for 10 min and the extent of hemolysis was determined by measuring the absorbance at 540 nm corresponding to hemoglobin liberation.

### 2.9. Cell lines and Growth Conditions

Human cancer cell lines, namely, lung (A549 and HOP-62), ovary (IGR-OV-1), and colon cancer (HCT-116), were procured from the National Center for Cell Sciences, Pune, India. Cell lines were grown and maintained in RPMI-1640 medium, pH 7.4 with 10% FCS, 100 units/mL penicillin, 100 *μ*g/mL streptomycin, and 2 mM glutamine. Cells were grown in CO_2_ incubator (Heraeus, GmbH, Germany) at 37°C in the presence of 90% humidity and 5% CO_2_.

### 2.10. Cytotoxic Assay by Sulforhodamine B Dye (SRB Assay)

The* in vitro* cytotoxicity of* P. hysterophorus* extracts was determined using sulforhodamine B (SRB) assay [26]. Cell suspension (100 *μ*L, 1 × 10^5^ to 2 × 10^5^ cells per mL depending upon mass doubling time of cells) was grown in 96-well tissue culture plate and incubated for 24 hours. Stock solutions of test extracts were prepared in DMSO and serially diluted with growth medium to obtain desired concentrations. 100 *μ*L test extract (100 *μ*g/well) was then added to the wells and cells were further incubated for another 48 h. The cell growth was arrested by layering 50 *μ*L of 50% TCA and incubated at 4°C for an hour followed by washing with distilled water and then air dried. SRB (100 *μ*L, 0.4% in 1% acetic acid) was added to each well and plates were incubated at room temperature for 30 min. The unbound SRB dye was washed with 1% acetic acid and then plates were air dried. Tris-HCl buffer (100 *μ*L, 0.01 M, pH 10.4) was added and the absorbance was recorded on ELISA reader at 540 nm. Each test was done in triplicate. The values are reported as mean ± SD of three replicates.

### 2.11. LC-MS Analysis of* P. hysterophorus* Extracts

LC-MS analyses of* P. hysterophorus* leaf (ET), stem (AC), flower (EA), and root (AC) extracts were performed on an Agilent Technologies 1200 series UPLC with 6540 series electrospray ionization (ESI), MS mode. Samples were dissolved in methanol and water (1 : 1 v/v). The separation was achieved using a Zorbax Eclipse XDB-C18 RR 4.6 mm × 50 mm × 1.8 *μ*m (Agilent Technologies) reversed-phase column held at 45°C. ESI parameters were as follows: nebulizer gas (N_2_) temperature 350°C; flow 10 L/min; pressure 40 psi; capillary voltage 4000 V. The fragmentor voltage was 135 V and injection volume for all samples was 5 *μ*L. The binary mobile phase consisted of water (solvent A) and acetonitrile (solvent B). Gradient elution was performed using the following solvent. Percentage of solvent B was 10, 10, 50, 90, 90, 50, and 10 % at 0.01, 2, 12, 22, 35, 45, and 50 min, respectively, at a flow rate of 0.7 mL/min.

### 2.12. Statistical Analysis

All experiments were carried out in triplicate and data were expressed as mean ± standard deviation (SD) or standard error of mean (SEM). The plots were prepared using GraphPad Prism software. Data were analyzed using one way ANOVA and the values of *P* < 0.05 were considered as statistically significant.

## 3. Results

### 3.1. Antibacterial Activity

The antibacterial activities of the extracts derived from leaves, stem, flower, and root of* P. hysterophorus* were evaluated against Gram positive* Streptococcus mutans* (MTCC 497) and Gram negative* Proteus vulgaris* (MTCC 7299) and* Salmonella typhi* (MTCC 3917) bacterial strains. The antibacterial activity profiles of* P. hysterophorus* extracts are given in Table 1.* P. hysterophorus* leaf extracts prepared in HX, BZ, and CH exhibited bacterial growth inhibition potential at 2 mg/disc concentration against* S. mutans, S. typhi,* and* P. vulgaris* with 12–20 mm ZOI against all the three test bacteria. However, inhibitory efficacy of leaf HX and BZ were more pronounced. ET extract of leaf showed moderate activity (ZOI 12 mm) against* S. mutans*. Among* P. hysterophorus* stem extracts nonpolar fractions showed moderate antibacterial activity (ZOI 10–12 mm) against* S. mutans* while ET extract showed considerable antibacterial activity (ZOI 17 mm).* S. typhi* exhibited resistance to all the stem extracts. No activity was observed with nonpolar extracts of stem against* P. vulgaris.* HX, BZ, EA, and AQ extracts of* P. hysterophorus* flower were active against* S. mutans.* Flower HX extract accounted for noticeable inhibitory activity (ZOI 16 mm) against* S. mutans.* Similarly, HX, CH, EA, and AQ fractions of* P. hysterophorus* root extracts showed antibacterial activity (ZOI 12–16 mm) against* S. mutans.* However,* S. typhi* and* P. vulgaris* exhibited complete resistance against flower and root extracts.

### 3.2. Total Flavonoid Content

Total flavonoid content in* P. hysterophorus* extracts are shown in Table 2. Differential amount of flavonoid contents were present in all the extracts. Comparatively higher amount of flavonoid content was found in* P. hysterophorus* leaf CH (14.55 ± 0.16 *μ*g QE/mg), stem ET (16.55 ± 0.21 *μ*g QE/mg), and flower CH, EA, and AC (36.29 ± 0.17, 59.62 ± 0.14, 41.41 ± 0.14 *μ*g QE/mg) extracts.* P. hysterophorus* flower fractions exhibited higher flavonoid content in the range of 13.90–59.62 ± 0.14 *μ*g QE/mg.

### 3.3. Phosphomolybdate Assay

**Figure 1:**
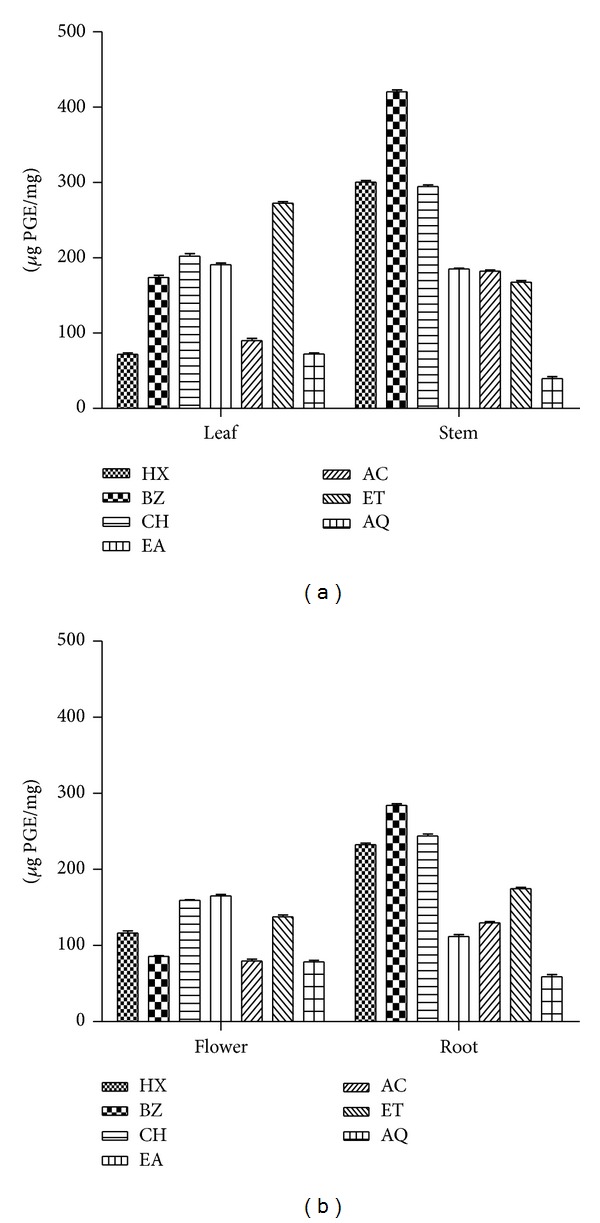
Total antioxidant activity of* P. hysterophorus* by phosphomolybdate assay: (a) leaf and stem and (b) flower and root extracts. Values are expressed as *μ*g propyl gallate equivalent/mg sample. HX: hexane; BZ: benzene; CH: chloroform; ET: ethyl acetate; AC: acetone; ET: ethyl alcohol; AQ: water. The results are expressed as mean ± SD (*n* = 3, *P* < 0.05).

The extracts derived from* P. hysterophorus* exhibited various degrees of antioxidant (AO) capacity (Figures 1(a) and 1(b)). Antioxidant capacity was in the range of 71–272 (leaf), 39–420 (stem), 78–165 (flower), and 58–284 (root) *μ*g PGE/mg of extract, respectively. Among all the test extracts of* P. hysterophorus* stem BZ fraction showed maximum antioxidant capacity (420 *μ*g PGE/mg).

### 3.4. Beta-Carotene Bleaching Inhibition Activity

**Figure 2:**
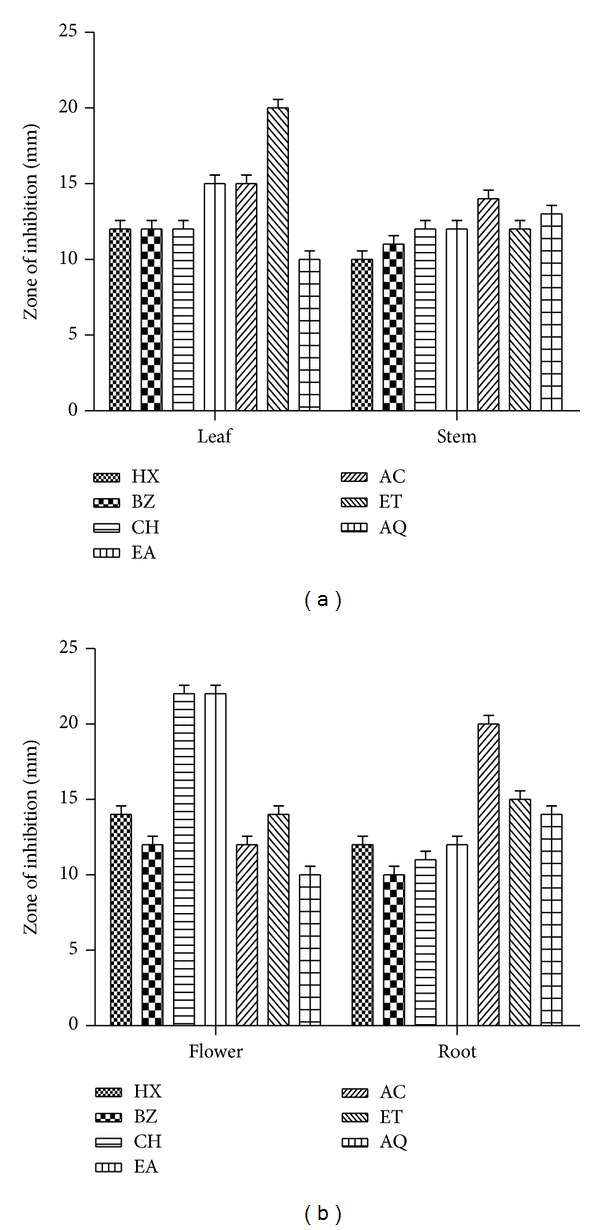
Beta-carotene bleaching inhibition activity of* P. hysterophorus:* (a) leaf and stem and (b) flower and root extracts. The values are represented as ZOI in mm. The results are expressed as mean ± SD (*n* = 3, *P* < 0.05). HX: hexane; BZ: benzene; CH: chloroform; EA: ethyl acetate; AC: acetone; ET: ethyl alcohol; AQ: water.

Antioxidant activity of the* P. hysterophorus* extracts was assayed by *β*-carotene bleaching well agar diffusion method and results are depicted in Figures 2(a) and 2(b). Leaf EA, AC, and ET fractions exhibited comparatively better antioxidant potential as indicated by larger ZOI 15-20 mm. All the stem extracts accounted for 10–14 mm ZOI (Figure 2(a)). Flower extracts (CH and EA) showed appreciable antioxidant activity (ZOI 22 mm) in Beta-carotene bleaching assay. Rest of the flower fraction exhibited antibleaching activity in the range of 10–14 mm ZOI. Some of the root extracts (AC, ET, and AQ) showed considerable activity (ZOI 14–20 mm). Rest of the root fractions exhibited moderate antioxidant activity (Figure 2(b)). The standard compound BHA showed ZOI-25 mm at 2 mg/mL concentration (not shown in figure).

### 3.5. Lipid Peroxidation Inhibition as a Marker of Biomembrane Protection

**Figure 3:**
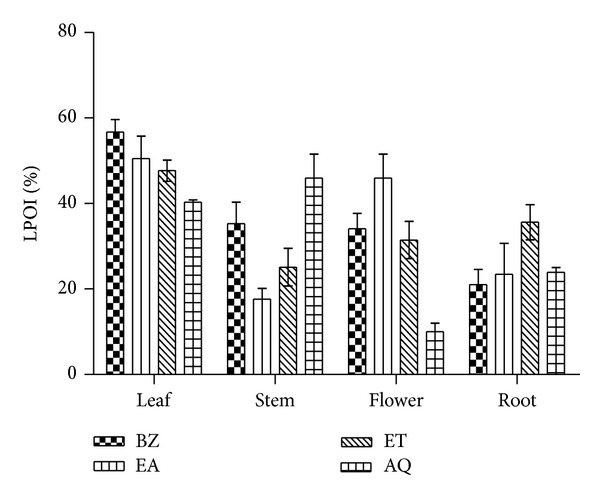
Lipo-protective efficacy of* P. hysterophorus* leaf, stem, flower, and root extracts in tissue (rat brain) homogenate. % LPOI (lipid peroxidation inhibition) activity of root extracts (BZ, EA, ET, and AQ) at a concentration of 0.2 mg/mL was assessed as an indicator to protect peroxidative damage of membrane lipids in rat brain homogenate. BHA (LPOI 80%) was used as control. The results are expressed as mean ± SD of three replicates (*P* < 0.05). BZ: benzene; EA: ethyl acetate; ET: ethyl alcohol; AQ: water.

*In vitro* membrane protective efficacy of* P. hysterophorus* extracts in rat brain tissue homogenate was assayed and % LPOI is shown in Figure 3. Among leaf extracts BZ fraction accounted for about 57% protection against membrane peroxidative damage while % LPOI observed with other extracts was in the range of 40–51%. Similarly AQ and EA fractions of stem and flower, respectively, showed about 46% antiperoxidation efficacy. The % LPOI for root and other extract fractions demonstrated low inhibitory activity (10–35%) against membrane damage in rat brain homogenate. BHA showed better protective response (LPOI about 80%, not shown in figure).

### 3.6. Antihemolytic Activity

**Figure 4:**
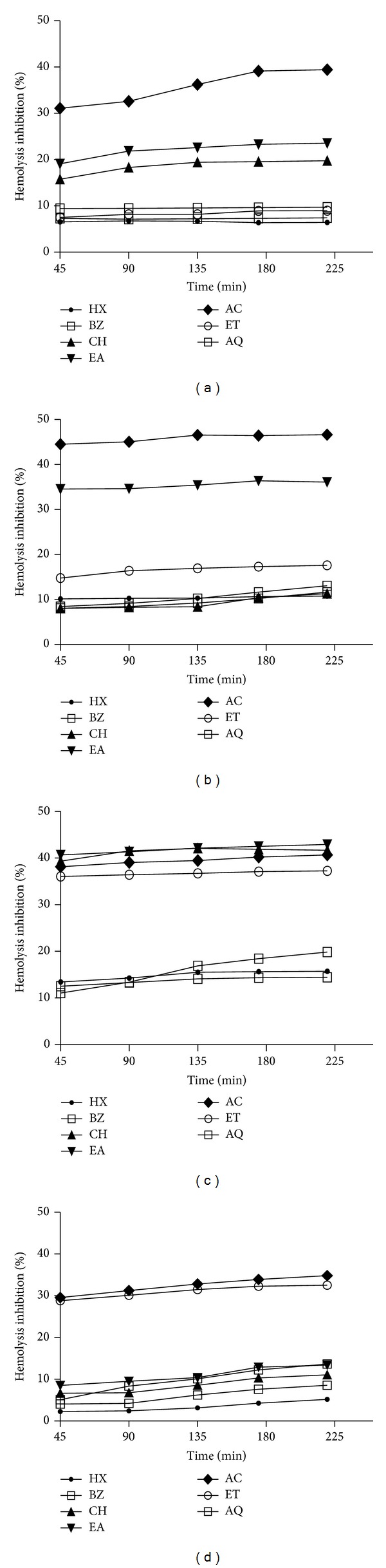
Antihemolytic activity of* P. hysterophorus:* (a) leaf, (b) stem, (c) flower, and (d) root extracts in rat red blood cells. The results are expressed as mean ± SD of three replicates (*P* < 0.05). HX: hexane; BZ: benzene; CH: chloroform; EA: ethyl acetate; AC: acetone; ET: ethyl alcohol; AQ: water.

Antihemolytic activity of* P. hysterophorus* extracts was evaluated in H_2_O_2_ induced rat blood erythrocyte membrane damage and the results are shown in Figures 4(a)–4(d). Some of the leaf (AC), stem (AC and EA), flower (CH, EA, AC, and ET), and root (AC and ET) extracts exhibited moderate antihemolytic activity (30%–45%). Rest of the test extract did not provide protection against H_2_O_2_ induced erythrocyte membrane damage.

### 3.7. Cytotoxic Assay by Sulforhodamine B Dye (SRB Assay)

**Figure 5:**
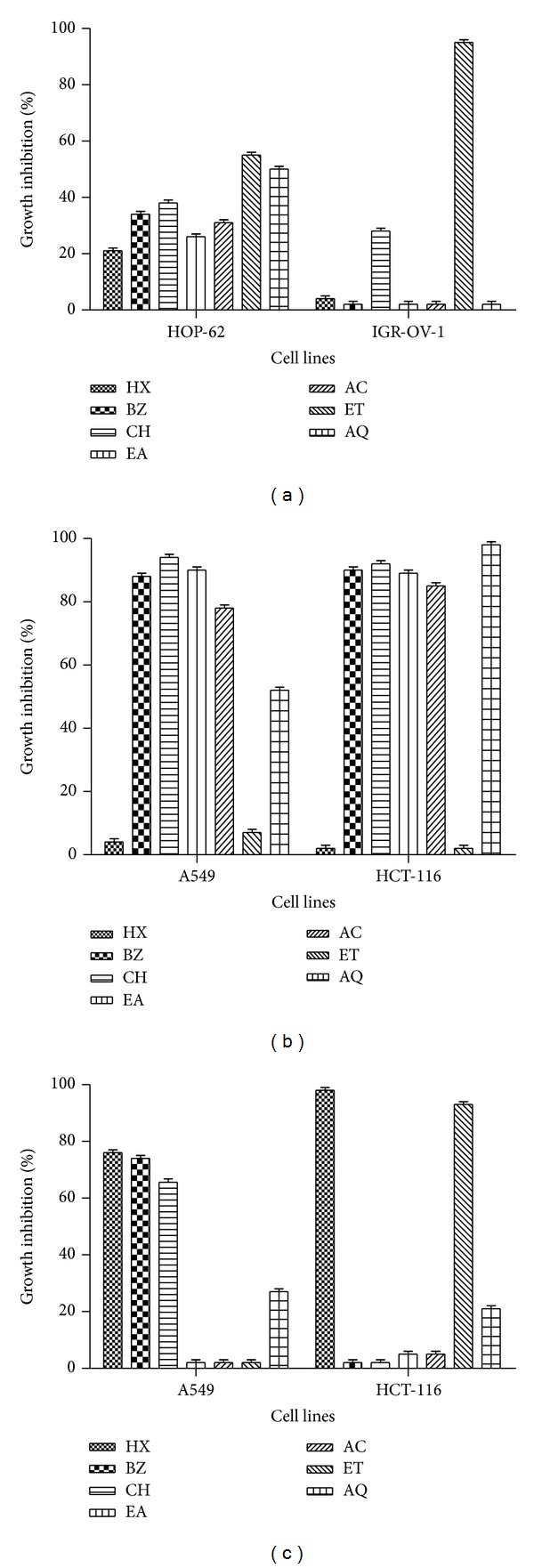
Cytotoxic effect of* P. hysterophorus:* (a) leaf, (b) flower, and (c) root extracts against cancer cell lines using SRB assay. Percentage growth inhibition of HOP-62 (lung), IGR-OV-1 (ovary), HCT-116 (colon), and A-549 (lung) cancer cell lines was assayed at 100 *μ*g/mL concentration of extracts as described in materials and methods section. HX: hexane; BZ: benzene; CH: chloroform; EA: ethyl acetate; AC: acetone; ET: ethanol; AQ: water. Data represent mean ± SD of three replicates (*P* < 0.05).

The cytotoxicity activity of* P. hysterophorus* leaf, flower, and root extracts was tested against different cancer cell lines using SRB assay at the concentration of 100 *μ*g/mL and results are shown in Figures 5(a)–5(c).* P. hysterophorus* leaf extracts were tested against HOP-62 (lung) and IGR-OV-1 (ovary) while cytotoxicity of flower and root fractions were investigated against A-549 (lung) and HCT-116 (colon) cancer cell lines. Among leaf extracts ET fraction showed comparatively better cytotoxic activity against test cell lines (Figure 5(a)). ET extract showed 95% and 55% growth inhibition against IGR-OV-1 and HOP-62 cell lines, respectively. Rest of the leaf fractions showed 2–50% cytotoxic activity against test cell lines. Most of the flower extracts accounted for appreciable inhibitory potential (Figure 5(b)). BZ, CH, EA, and AC fractions of flower demonstrated 84–92% cytotoxic activity against A549 and HCT-116 cell lines. AQ fraction showed 100% cytotoxicity against HCT-116 cell line. Among* P. hysterophorus* root extracts HX and ET also showed appreciable activity (99% and 93%, resp.) against HCT-116 cells (Figure 5(c)). HX, BZ, and CH fractions demonstrated 76%, 74%, and 65% toxicity against A549 cell lines, respectively. Rest of the fractions accounted for low activity.

### 3.8. LC-MS Analysis

LC-MS analysis of the potent* P. hysterophorus* extracts revealed the presence of various compounds having different molecular weight. The LC-MS scan demonstrated presence of different compounds showing 3–20 min retention time (RT). The scan showing different RT of the compounds present in extract samples is represented in Supplementary Figures 1(a)–1(d) (see the Supplementary Material available online at http://dx.doi.org/10.1155/2014/495154). The RT, peak area, and* m/z* ratio of most abundant peaks are shown in Supplementary Table 1.

## 4. Discussion

Emergence of multiple drug resistance in human pathogenic organisms has given momentum to search new antimicrobial substances from alternative sources. There have been several mechanisms proposed for the antibacterial activity of potent drugs including plant extracts [21]. In many cases phytochemicals can be more effective than chemically synthesized pure compounds because they are a complex mixture of components. Their complexity enables them to interact with multiple molecular targets and thus it becomes more difficult for target microorganisms to develop resistance because of multiple response sites [27]. Some of the test extracts in the current work exhibited considerable antibacterial activity. Nonpolar extracts (HX and BZ) of* P. hysterophorus* leaf showed appreciable activity (ZOI 14–20mm) against* S. mutans, S. typhi, *and* P. vulgaris* (Table 1). The literature revealed that secondary metabolites such as alkaloids, tannins, flavonoids, and other phytochemicals are responsible for the antimicrobial activities in higher plants [16, 28]. It is possible that flavonoids in* P. hysterophorus* extracts and other group of phytochemicals as reported in our previous study [2, 4] may find their use as future antibacterial agents.

In phosphomolybdenum assay antioxidants reduce molybdenum (VI) to green coloured molybdenum (V) complex. The molybdenum (V) complex shows absorption maxima at 695 nm [29]. Most of the extracts obtained from* P. hysterophorus* stem showed appreciable antioxidant activity (Figures 1(a) and 1(b)). The difference in AO capacity of different extracts may be attributed to differences in their chemical composition. The antioxidant activities of the individual phenolic compounds may depend on structural factors, such as the number of phenolic hydroxyl or methoxyl groups, flavone hydroxyl, keto groups, free carboxylic groups, and other structural features. Recent reports indicated that several bioactive compounds present in plants have strong antioxidant activity [18, 21]. Chromophores such as *β*-carotene have alternate double and single carbon-carbon bonds which are known as conjugated system. The electrons in the *π*-orbitals of the double bonds overlap, creating a system of delocalized electrons across a large part of the molecule. Carotenoids undergo bleaching (loss of color) when exposed to radicals or to oxidizing species which involves interruption of the conjugated double bond system either by cleavage or by addition to one of the double bonds [30]. The results demonstrated that some of the leaf (ET), flower (CH and EA), and root (AC) extracts possess appreciable *β*-carotene bleaching inhibition activity (<20 mm ZOI). This indicates that* P. hysterophorus* extracts exhibit antioxidant potential by virtue of their radical scavenging activity. Polyphenolic contents of the extracts have been reported to function as good electron and hydrogen atom donors and therefore should be able to terminate radical chain reaction by converting free radicals and ROS to more stable products [2, 14, 31].

ROS produces a broad spectrum of responses based on the magnitude of the level, duration of exposure, its localization, and nature. At higher levels, it can easily react with membrane lipids, DNA, and proteins [31, 32]. This interaction results into alteration of membrane permeability, damages genomic stability by causing oxidative modifications, influences catalytic activity of enzymes, and/or make proteins more susceptible to proteolytic degradation. Moreover, through distinct signal transduction cascades, ROS can induce the expression of families of heat shock proteins and antioxidative enzymes which help to regulate redox homeostasis [1]. In present study we used* in vitro* iron induced lipid-peroxidation model to assess the membrane protective efficacy of* P. hysterophorus* extracts in albino Wistar rat brain tissue homogenate. Some of the test extracts showed moderate (up to 50%) lipo-protective activity which may be attributed to their flavonoid content [8]. Hemolysis has long been used to measure free radical damage and its inhibition by antioxidants in whole blood erythrocytes. This assay is useful for screening of various chemical compounds and plant extracts having antioxidant potential [33]. In present study we used rat blood erythrocytes to study H_2_O_2_ induced membrane damage. Lipid oxidation of rat blood erythrocyte membrane mediated by H_2_O_2_ induces membrane damage and subsequently hemolysis occurs.* P. hysterophorus* extracts showed moderate antihemolytic activity (Figures 4(a)–4(d)). Several studies have revealed moderate to higher efficacy of plant products against lipid oxidation in the erythrocytes membranes [33, 34].

From the year 1981–2002 reports showed that approximately 60% of anticancer agents are derived from natural products. Herbal drugs do not only serve as drugs but also provide a rich source of novel structures that may be developed into novel anticancer agents [35]. Present study demonstrated that some of the* P. hysterophorus* extracts exhibited appreciable anticancer activity (80–100%) against ovary, lung, and colon cancer cell lines (Figure 5). Most of the flower extracts (BZ, CH, EA, AC, and AQ) showed 78%–98% cell growth inhibition of lung and colon cancer cells. HX and ET extract of root exhibited up to 98% cytotoxicity against colon cell line. Most of the potent cytotoxic extract (Figure 5) possess higher amount of flavonoid contents (Table 2). Thus, it may be inferred that the presence of flavonoid in the extracts is mainly responsible for the significant anticancer activity of* P. hysterophorus* extracts. Many other studies have revealed the chemopreventive role of flavonoids in cancer through their effects on signal transduction in cell proliferation and angiogenesis [31]. In addition a number of other mechanisms are also involved in the process. Kumar and Pandey [8] have described the mechanisms by which flavonoids can exert their anticancer activity. LC-MS analysis of the* P. hysterophorus* leaf (ET), stem (AC), flower (EA), and root (BZ) extract showed variation in separated compounds as indicated by different RT, peak area, and* m/z* values (Supplementary Figures 1(a)–(d) and Supplementary Table 1). Occurrence of the variable pattern of distribution of compounds may be responsible for the different extent of biological activities shown by the test extracts.

## 5. Conclusion

The study revealed that phytochemicals present in various parts of the* P. hysterophorus* extracts exhibit biological properties. Compounds present in leaf have antibacterial activity. Many extract fractions of leaf, flower, stem, and root extracts exhibited antioxidant and cytotoxic potential. LC MS data indicated presence of many compounds in the extracts with different RT (3–20 min).

## Supplementary Material

Supplementary Figures 1(a)-1(d) depict LC-MS chromatogram showing retention time (RT) and relative abundance of various compounds present in potential extracts of different parts of *P. hysterophorus*. Data shown in supplementary Table 1 indicate the RT, peak area and m/z ratio of most abundant peaks in potential extracts as observed in supplementary Figure 1 for *P. hysterophorus* leaf, stem, flower, and root. The detailed methodology of LC-MS analysis is described in Materials and Methods Section (2.11) of the main article.

## Figures and Tables

**Table 1:** Antibacterial activity of *P. hysterophorus* extracts.

Extracts	*S. mutans *				*S. typhi *				*P. vulgaris *			
	Leaf	Stem	Flower	Root	Leaf	Stem	Flower	Root	Leaf	Stem	Flower	Root
HX	14	11	16	12	16	—	—	—	16	—	—	—
BZ	16	12	12	10	20	—	—	—	20	—	—	—
CH	12	11	10	14	12	—	—	—	12	—	—	—
EA	09	10	12	16	—	—	—	09	—	—	10	—
AC	—	11	—	09	—	—	—	—	—	12	—	—
ET	12	17	—	—	—	09	—	—	—	10	—	—
AQ	—	12	13	16	—	10	10	09	—	12	10	—

Zone of inhibition (ZOI) values are reported as average of three replicates. The extract contents present in the discs were 2 mg/disc. HX: hexane; BZ: benzene; CH: chloroforms; EA: ethyl acetate; AC: acetone; ET: ethyl alcohol; AQ: water.

**Table 2:** Flavonoid content in *P. hysterophorus *extracts.

Extracts	Leaf	Stem	Flower	Root
HX	10.93 ± 0.23	11.13 ± 0.15	13.90 ± 0.03	6.42 ± 0.05
BZ	7.60 ± 0.15	14.31 ± 0.22	15.01 ± 0.08	4.99 ± 0.09
CH	14.55 ± 0.16	5.42 ± 0.18	36.29 ± 0.17	7.36 ± 0.11
EA	10.83 ± 0.25	4.62 ± 0.13	59.62 ± 0.14	2.95 ± 0.13
AC	10.05 ± 0.18	3.38 ± 0.11	41.41 ± 0.14	4.58 ± 0.18
ET	6.66 ± 0.21	16.55 ± 0.21	27.43 ± 0.10	1.98 ± 0.01
AQ	2.64 ± 0.20	2.58 ± 0.09	20.25 ± 0.12	1.88 ± 0.02

The values are represented as *μ*g quercetin equivalent per milligram of sample (*μ*g QE/mg). The results are expressed as mean ± SEM (*n* = 3). HX: hexane; BZ: benzene; CH: chloroform; EA: ethyl acetate; AC: acetone; ET: ethyl alcohol; AQ: water.
