# Supplementary material for: In Vitro Antibacterial, Antioxidant, and Cytotoxic Activities of Parthenium hysterophorus and Characterization of Extracts by LC-MS Analysis
Source: Biomed Res Int. 2014 May 7;2014:495154. doi: 10.1155/2014/495154 (PMC4033558; doi:10.1155/2014/495154)
Supplement: Supplementary file 1 — Supplementary Figures 1(a)-1(d) depict LC-MS chromatogram showing retention time (RT) and relative abundance of various compounds present in potential extracts of different parts of P. hysterophorus. Data shown in supplementary Table 1 indicate the RT, peak area and m/z ratio of most abundant peaks in potential extracts as observed in supplementary Figure 1 for P. hysterophorus leaf, stem, flower, and root. The detailed methodology of LC-MS analysis is described in Materials and Methods Section (2.11) of the main article. [file 495154.f1.docx]

**
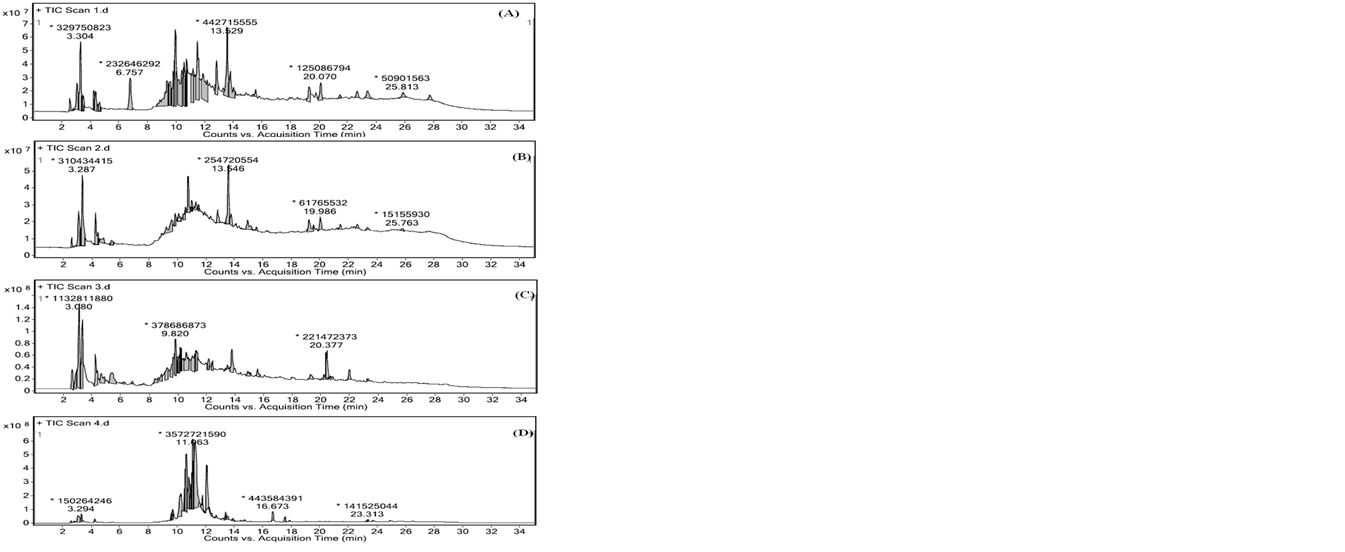
**

**Supplementary Figure 1: LC-MS chromatogram showing retention time (RT) and abundance of various compounds present in *P. hysterophorus* (A) leaf-ET, (B) stem-AC, (D) root-BZ, and (D) flower-EA extracts.** Abbreviations: ET-ethyl acetate, AC-acetone, EA-ethyl acetate and BZ-benzene.

**Supplementary Table 1: LC-MS data for *P. hysterophorus* extracts**

| Extract | Retention  time (min) | Peak area | Precursor  Ion (m/z) |
| --- | --- | --- | --- |
| Leaf (ET) | 3.304 | 205056708 | 146.1170 |
|  | 6.757 | 207013094 | 116.0857 |
|  | 9.911 | 214429215 | 462.2323 |
|  | 11.4 | 177688185 | 249.1479 |
|  | 12.799 | 147526490 | 347.0754 |
|  | 13.529 | 295654404 | 282.1690 |
| Stem (AC) | 3.287 | 203341658 | 116.1066 |
|  | 4.217 | 56991028 | 130.0493 |
|  | 10.724 | 108951156 | 295.2846 |
|  | 13.546 | 200940459 | 280.1537 |
|  | 19.190 | 50910583 | 439.3550 |
|  | 19.986 | 60836885 | 316.2833 |
| Flower (EA) | 10.233 | 1623445007 | 868.5139 |
|  | 10.631 | 1693963494 | 576.3910 |
|  | 11.063 | 1523269145 | 414.3417 |
|  | 11.229 | 1778503043 | 868.5694 |
|  | 12.026 | 2871727320 | 722.4948 |
|  | 16.673 | 504803442 | 414.3435 |
| Root (BZ) | 3.080 | 611208981 | 236.1678 |
|  | 3.313 | 364498973 | 118.0862 |
|  | 4.209 | 161486615 | 146.117 |
|  | 5.404 | 214195448 | 158.1169 |
|  | 9.820 | 170632150 | 388.2529 |
|  | 13.737 | 208767989 | 439.3557 |
|  | 20.377 | 314697522 | 286.1432 |

Analysis conditions: Acquisition mode-MS; Ion polarity-positive; Species-[M+H]^+^; Fragmentor voltage- 135 V
